# Identification of genomic biomarkers and their pathway crosstalks for deciphering mechanistic links in glioblastoma

**DOI:** 10.1049/syb2.12066

**Published:** 2023-06-05

**Authors:** Darrak Moin Quddusi, Naim Bajcinca

**Affiliations:** ^1^ Chair of Mechatronics in the Faculty of Mechanical and Process Engineering Rheinland‐Pfälzische Technische Universität Kaiserslautern‐Landau Kaiserslautern Germany

**Keywords:** bioinformatics, cancer, genomics

## Abstract

Glioblastoma is a grade IV pernicious neoplasm occurring in the supratentorial region of brain. As its causes are largely unknown, it is essential to understand its dynamics at the molecular level. This necessitates the identification of better diagnostic and prognostic molecular candidates. Blood‐based liquid biopsies are emerging as a novel tool for cancer biomarker discovery, guiding the treatment and improving its early detection based on their tumour origin. There exist previous studies focusing on the identification of tumour‐based biomarkers for glioblastoma. However, these biomarkers inadequately represent the underlying pathological state and incompletely illustrate the tumour because of non‐recursive nature of this approach to monitor the disease. Also, contrary to the tumour biopsies, liquid biopsies are non‐invasive and can be performed at any interval during the disease span to surveil the disease. Therefore, in this study, a unique dataset of blood‐based liquid biopsies obtained primarily from tumour‐educated blood platelets (TEP) is utilised. This RNA‐seq data from ArrayExpress is acquired comprising human cohort with 39 glioblastoma subjects and 43 healthy subjects. Canonical and machine learning approaches are applied for identification of the genomic biomarkers for glioblastoma and their crosstalks. In our study, 97 genes appeared enriched in 7 oncogenic pathways (RAF‐MAPK, P53, PRC2‐EZH2, YAP conserved, MEK‐MAPK, ErbB2 and STK33 signalling pathways) using GSEA, out of which 17 have been identified participating actively in crosstalks. Using PCA, 42 genes are found enriched in 7 pathways (cytoplasmic ribosomal proteins, translation factors, electron transport chain, ribosome, Huntington's disease, primary immunodeficiency pathways, and interferon type I signalling pathway) harbouring tumour when altered, out of which 25 actively participate in crosstalks. All the 14 pathways foster well‐known cancer hallmarks and the identified DEGs can serve as genomic biomarkers, not only for the diagnosis and prognosis of Glioblastoma but also in providing a molecular foothold for oncogenic decision making in order to fathom the disease dynamics. Moreover, SNP analysis for the identified DEGs is performed to investigate their roles in disease dynamics in an elaborated manner. These results suggest that TEPs are capable of providing disease insights just like tumour cells with an advantage of being extracted anytime during the course of disease in order to monitor it.

## Graphical abstract

We used gene expression data from TEPs for glioblastoma and analysed it using GSEA and PCA to quantify differentially expressed genes. We further identified crosstalks between enriched pathways that foster cancer hallmarks, followed by SNP analysis of DEGs.

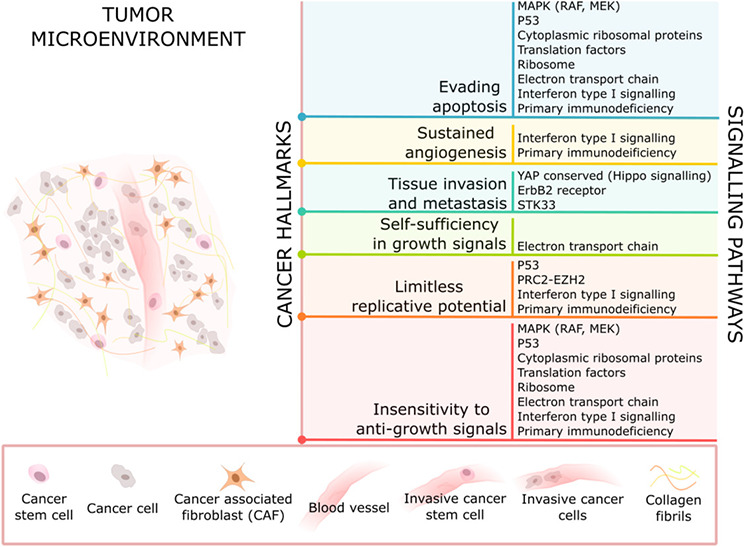

## 1 INTRODUCTION

Glioblastoma is the most belligerent brain tumour that occurs in glial cells, rarely spreading to distant organs. Though glial cells do not participate in organism‐wide communication like neurons but they play equally significant roles by providing support to neurons, [1] and thus, anomalies in glial cells can lead to havoc also. Glioblastoma is a rapidly growing lethal brain tumour that has been declared as grade IV astrocytoma by WHO (World Health Organisation) [2]. It often occurs in the cerebral hemispheres mostly temporal and frontal lobes of brain [3]. It has an incidence rate of 3.19 glioblastoma cases per 100,000 persons every year [4]. Once diagnosed, the average life expectancy is approximately 12–15 months, 25% of patients survive for about a year while less than 5% have a life expectancy of 5 or more years [5].

Glioblastoma actively proliferates and in order to stop it from growing further, surgery appears as an approachable solution; but since it insinuates the surrounding tissue, complete resection is not possible. In such conditions, radiotherapy does not give good results either [6] because of blood‐brain barrier, which renders treatment difficult as tumour cells present in the hypoxic regions show resistance to radiotherapy [7]. Thus, the possible effective treatment is to abscise the tumour as much as possible followed by chemo and radiotherapy [8]. Treatment can add up to a few months in the life expectancy of a glioblastoma patient.

Biomarkers serve as quantifiable indicators of perturbed biological processes, which appear differently in normal processes [9]. They help in understanding the spectrum of disease dynamics, which can further aid in either eradicating the disease‐causing disturbance or minimising the disease spread. Thus, the identification of biomarkers is very important as they assist in tracking the record from diagnosis of a disease to its treatment and post‐treatment symptoms in order to curtail the recurrence of disease. Moreover, they are very helpful in oncogenic decision‐making.

Glioblastoma is a very lethal cancer, and due to its smaller life expectancy, the patient dies even before its mystery is unwound. Brain tissue cannot be abscised from the body usually to perform studies because brain surgeries and biopsies are intracranial [4]. Since the causes are unknown for glioblastoma, genomic biomarkers hold a significant value in understanding the onset and prevalence of disease. As the human genome has been sequenced and high‐throughput sequencing techniques are producing bulks of genomics data, the identification of differentially expressed genes (DEGs) in glioblastoma patients can shed light on pre‐sequenced genes that have never been studied before with respect to the disease [10]. Causes and biomarkers of the disease vary from individual to individual but in order to make generalised inferences; a combined study of gene expressions in various individuals is helpful to identify which group of DEGs is significantly important. Therefore, it necessitates the identification of genomic biomarkers for glioblastoma using different population data to provide some more generalised information in order to understand the onset of disease and to find new approaches for treatment so that life expectancy can be increased. The most well‐studied and known genomic biomarkers for glioblastoma noted so far are as follows: EGFR, PTEN, TERT, FGFR2, IRS2, AKT3, TP16, TP53, PARK2, PTPRD, NF1, IDH1, PDGFRA, and STAT3 [4, 10, 11] associated with amplifications, mutations, and loss of heterozygosity.

The identification of genomic biomarkers is conventionally performed using tissue biopsies to characterise different tumours and their types. This approach renders limited samples and incompletely represents tumours as it cannot be recursed to monitor the disease. Therefore, liquid biopsies (aka blood‐based liquid biopsies have been found niftier due to their non‐invasive nature and ability to provide maximal tumour information) [12]. They aid in generating information at transcriptomic, proteomic, epigenomic, and metabolomic levels. Blood serves as a wealth of information as it contains numerous analytes, that is, circulating tumour cells (CTCs), circulating tumour DNA (ctDNA), tumour‐educated platelets (TEPs), and extracellular vesicles (EVs), such as exosomes, metabolites, and proteins [13].

Biological systems are very complex in nature and biological pathways work in a coordinated manner to generate appropriate responses to perturbations from internal or external stimuli. Therefore, the interaction of genes involved in an underlying physiological system may reveal valuable information at a mechanistic level to increase our understanding of complex molecular dynamics. Besides gene expression profiles, the interaction of these genes in their regulatory networks unleashes valuable information as to the way they play their biological role [14]. Therefore, to comprehend cellular behaviour under certain conditions, it is helpful to identify these interactions and crosstalks.

### 1.1 Motivation and contribution

This work aims at providing potential genomic biomarkers for glioblastoma and their crosstalks between cancer‐fostering pathways to improve the understanding of disease dynamics. Therefore, well‐established canonical and machine‐learning approaches have been used to carry out this study. This study utilises blood‐based liquid biopsies primarily focusing on tumour‐educated platelets (TEPs). Duly mentioned above biomarkers from blood‐based liquid biopsies hold the potential to capture cancer signatures at the genomic level. In the past, genomic biomarkers for glioblastoma have been identified using tumour biopsies [15, 16, 17, 18], but these studies exist as small pieces of a puzzle as vast biodiversity lies at the population level based on ethnicity, age, sex, and continuously varying environmental exposure. Our study will also serve as a piece in the puzzle of glioblastoma dynamics in order to acquire mechanistic insights. Moreover, SNP‐based associations with various brain disorders have further helped in scrutinising the roles of these biomarkers in glioblastoma. To the best of our knowledge, there does not exist any study where TEPs are used to identify genomic biomarkers and their crosstalks in tumour‐harbouring pathways for glioblastoma.

The paper is organised as follows: Section 2 describes the description and preprocessing of data, dimensionality reduction, and classification problems followed by enrichment analysis. Section 3 addresses the results of the study. The significance of results is discussed in detail in Section 4 and Section 5 marks the conclusion of the paper.

## 2 METHODS

**FIGURE 1:**
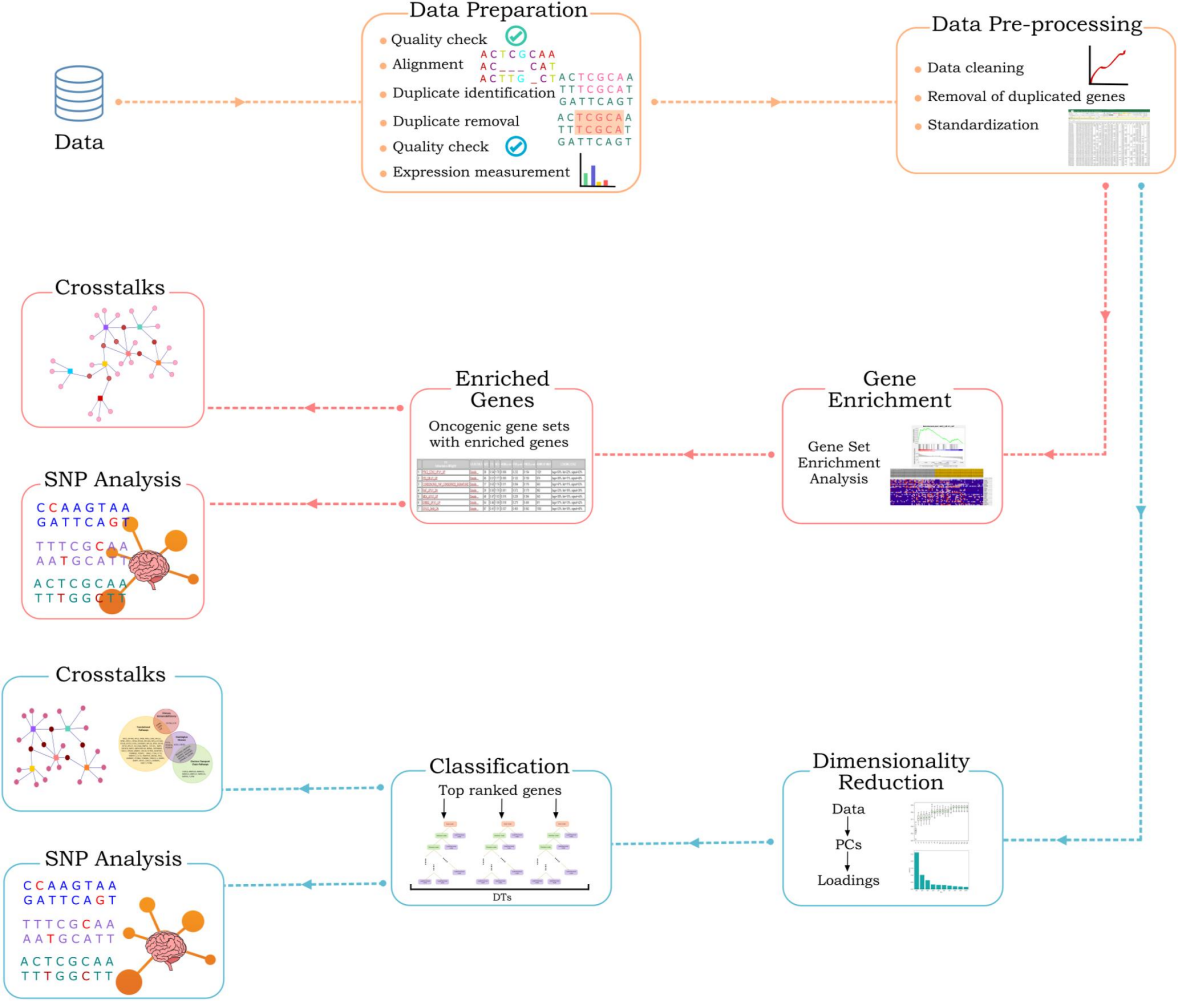
Workflow of the current study protocol. To attain the objectives of this study, raw gene expression data from TEPs in the form of RNA‐seq for glioblastoma is used; followed by its preparation and preprocessing it underwent two distinct approaches, namely GSEA and PCA. GSEA and PCA‐derived DEGs and enriched pathways were subjected to pathway analysis in order to study the involvement of DEGs in crosstalks between enriched pathways that foster cancer hallmarks, followed by SNP analysis of DEGs.

This section describes the protocol adopted for the current study. Figure 1 gives an overview of the workflow. The integrants of this figure are explained in detail.

### 2.1 RNA‐seq data

mRNA expression profiles are downloaded from ArrayExpress with accession number GSE68086. ArrayExpress stores data generated from high‐throughput sequencing experiments. The inclusion criteria for dataset consider a human origin and it being free from mutations and drug effects. Unfortunately, not much data is available for glioblastoma because of its intracranial locus. Moreover, we wanted to work with liquid biopsies data; therefore, the current dataset has been chosen after a thorough screening of public databases keeping under consideration the criteria for dataset selection. This data had been obtained primarily from tumour‐educated platelets (TEP) using blood‐based liquid biopsies [19]. These expression profiles contained 39 experimental samples and 43 control samples. Details of selected samples are provided in Supporting Information S1 Table

### 2.2 Data preparation and preprocessing

RNA sequencing methods generate a lot of raw data, which needs to be processed prior to its use in order to discard unnecessary adaptor sequences and redundant gene transcripts, which if not removed can compromise the quality of results [20]. Preprocessing of data includes basic steps, such as quality control, read alignments, and quantification of genes and transcript levels mainly. There is a wide variety of available tools for this purpose [21]. We have used FastQC [22] for quality control, HISAT2 [23] for read alignment, and RSeQC [24] for identification of duplicate reads on the basis of sequence and mapping. MarkDuplicate and RmDup [25] are used to mark and remove the replicated sequences having the highest mapping quality, respectively. For the estimation of gene expression levels in aligned data files, StringTie [26] is used.

### 2.3 Machine learning

The coding scripts used in this section are available in Supporting Information S0 Scripts.

#### 2.3.1 Dimensionality reduction

High‐throughput sequencing yields high‐dimensional genomic data, which ultimately increase its volume [27]. Extracting information from such a high‐dimensional data is difficult; therefore, the information at the genomic level is often incomplete because it is not easy to extract the maximum information from available genomic signatures. Therefore, on such kind of high‐dimensional data, dimensionality reduction is performed to decrease the dimensions and volume of data, preserving the important signatures of data, which can explain the rest of data at a maximal level. Moreover, apart from information processing, dimensionality reduction is useful to improve the accuracy of classifiers for prediction in turn decreasing the computation cost yielding optimised results [28]. Dimensionality reduction helps in getting quality features for classification and regression by eliminating noise and redundant features. It reduces multicollinearity resulting in model's better performance. In this study, principal component analysis is used to cater the curse of dimensionality, which comes with genomic data.

In this study, principal component analysis (PCA) [29] with singular value decomposition (SVD) is used as a dimensionality reduction approach on the RNA‐seq dataset of glioblastoma. It is an unsupervised method that works by reducing the multicollinearity in data resulting in the formation of new features known as principal components, which are least correlated with each other. Let the dataset be a matrix *D* of *m* × *n* dimensions where *m* represents the number of samples and *n* represents the number of features (genes). Performing SVD on D [30]: *D* = *U*Σ*V* ^*T*^, where U represents a unitary matrix with *m* × *m* dimensions and Σ is a diagonal matrix of singular values that is, *σ*_*i*_ with *m* × *n* dimensions. Right singular vectors *V* depict principal directions and singular values *σ*_*i*_ are related to the eigenvalues *λ*_*i*_ as $\lambda_{i}=\sigma_{i}^{2}$. Principal components are calculated from *DV* = *U*Σ*V* ^*T*^ *V* = *U*Σ, and columns of *U*Σ represent principal components. Eigenvalues *λ*_*i*_ show variations captured by principal components in data. Whereas loadings are obtained from $\frac{V\Sigma }{\sqrt{m-1}}$.

#### 2.3.2 Classification

Decision tree is an ensemble learning method that falls under the category of supervised machine learning algorithms. It is used for both regression and classification problems. A decision tree represents the knowledge extracted from dataset in its inductive learning process where it splits the given dataset and creates a decision boundary between labels. It has three types of nodes; a “root node” from where it starts which has no incoming edge, “internal or test node” having both incoming and outgoing edges, and “leaf or terminal node”, which has no outgoing edge [31].

Splitting of data occurs at test nodes under a certain criterion to make the split optimum. In this study, entropy has been used as the criterion to measure the quality of the split. In simple words, entropy measures the uncertainty of an arbitrary variable, characterising the impurity of a randomly selected subset from sample space [32]; entropy can be calculated as follows:

$$H\left(S\right)=-\Sigma_{i}^{c}P_{i}\log_{2}P_{i}.$$

The next important step is attribute selection, which is determined by information gain. It is impurity‐based measure, which makes use of entropy [32]. Information gain can be calculated as follows:

$$\text{Gain}\left(\text{S,A}\right)=H\left(S\right)-\Sigma_{v\in Value\left(A\right)}\frac{\left|S_{v}\right|}{\left|S\right|}H\left(S_{v}\right),$$

where *S* is the set at parent node, *A* is the attribute, *Value*(*A*) is the set containing all values for *A*, *S*_*v*_ is the subset of *S* at the child node for which attribute *A* has value of *v*, that is, *S*_*v*_ = {*S*|*A*(*s*) = *v*}, and *C* = {0, 1}.

Usually, each test node considers one attribute in order to partition the sample space as per attribute's value. Terminal nodes also known as decision nodes represent the class labels. As decision tree works on the top‐down approach, samples are navigated from the root node to decision nodes of the tree in order to be classified. This classification path of samples is determined by the outcome of test nodes [31].

In this study, we have performed classification on the loading matrix of genes with resect to First three principal components to verify the results of dimensionality reduction. Out of several performance measures, we have used confusion matrix and accuracy to measure the performance of decision tree. Confusion matrix is the representation of true positive (TP), true negative (TN), false positive (FP), and false negative (FN) parameters in the form of a matrix. True positives and true negatives are those outcomes, which are classified correctly as per given condition when it exists and when it does not exist, respectively. Contrary to this false positives and false negatives are the misclassified outcomes. In false positives, outcomes are classified predicting that the condition exists when in actual it does not; in short rejecting the true null hypothesis. This misclassification error is known as type I error. In false negatives, outcomes are classified predicting that the condition does not exist when in actual it does; concisely, it is the failure to reject the false null hypothesis. This misclassification error is known as type II error [33]. Accuracy is another measure to assess the performance of classification models and it is computed on the basis of above mentioned four parameters (TP, TN, FP, and FN) [34] as follows:

$$\text{Accuracy}=\frac{\text{TN}+\text{TP}}{\text{TN}+\text{FP}+\text{TP}+\text{FN}}.$$

A detailed analysis on the selection of decision tree for this study is provided in Supporting Information S2 Analysis.

### 2.4 Enrichment analysis

The identification of DEGs for a certain disease is insufficient to utmostly interpret the genomic signatures lying in the dataset in turn thwarting the comprehension of disease dynamics. Therefore, enrichment analysis is used alongside to supplement the interpretation of gene expression data [35]. It is performed by focalising those groups of genes, which perform similar biological functions, share common chromosomal location, or regulate same biological pathways. These groups of genes are termed as gene sets [36]. Enrichment analysis is performed by ordering the genes with respect to their differential expression in both control and experimental groups in the form of a ranked list. This ranked list is used to identify the DEGs enriched in certain pathways, thus highlighting pathways that have been altered in the disease [36].

In this study, we have used gene set enrichment analysis (GSEA) [36] to interpret gene expression data as a canonical method. GSEA works by ranking the genes on the basis of correlation between their expressions with two phenotypes (experimental or control) in the form of a list. It is backed up with a database of gene sets, and the prime objective is to determine if the genes from gene sets appear enriched in the gene expression data or not. This is done by calculating an enrichment score, which is a measure of representation of genes from a gene set in the ranked list either at its top or the bottom as these regions clearly distinguish between two phenotypes. Statistical significance of enrichment score is estimated by producing a null distribution using a permutation test based on the phenotypes followed by the computation of nominal *p*‐value relative to the null distribution. In case of multiple gene sets involvement, estimated significance is readjusted to reckon multiple hypothesis testing. Thus, for each gene set, enrichment score is normalised and its false discovery rate is estimated [36].

### 2.5 Single nucleotide polymorphism (SNP) analysis of identified DEGs

We have performed single‐nucleotide polymorphism analysis on the DEGs, which we identified using PCA and GSEA. These DEGs comprise a total of 139 genes; 42 from PCA and 97 from GSEA. SNP analysis helps to further understand the behaviour of our identified DEGs as their variants appear as causative agents in numerous nervous disorders/diseases. Here, our concerned diseases belonged to the nervous system. This analysis has been performed using DisGeNET [37] version 7.0, which enables us to look up associations between genes and their variants with diseases. The gene names were provided as input in the search panel and top 5 variant‐disease associations were consulted for each gene considering the highest variant‐disease association score for brain disorders only. This variant‐disease association score shows the strength of association reckoning with the type and number of sources and publications, which support the association. It ranges from 0 to 1.

### 2.6 Crosstalks

Crosstalks between two or more pathways occur when some genes from one pathway are regulated in two or more respective pathways. The activation and deactivation of these genes not only affect one pathway but all the other pathways in which they are involved. Crosstalks are important to study as they provide a holistic view to understand the dynamics of biological processes, which are sometimes difficult to interpret when studying only one pathway [14]. Crosstalks for glioblastoma are generated using Cytoscape version 3.8.2 [38]. In this study, we have identified crosstalks between cancer fostering pathways where DEGs from both approaches (PCA and GSEA) are actively involved. These pathways are taken from two databases namely kegg and wiki pathways. Wikipathways [39] plugin was used to extract enriched pathways, whereas enriched pathways from kegg were downloaded from kegg pathway database and gene names were extracted.

## 3 RESULTS

This section comprises of glioblastoma DEGs and their crosstalks, which we have identified using PCA as a non‐canonical and gene set enrichment analysis as a canonical method.

### 3.1 Dimensionality reduction and clustering

In this study, we had 9797 genes as features after data preparation and preprocessing steps. We reduced this number to 6126 by removing the redundant genes, that is, transcripts of the same gene, by selecting the high‐intensity gene transcript and preserving only their viable expression values. We applied PCA on these 6126 genes to further reduce the dimensions of dataset in order to capture the signature of those genes, which were capable of classifying the experimental and control samples. In this regard, first three principal components were selected as these were capturing the 42% of variance in the dataset. The variance captured by first three components is 26%, 10%, and 6%, respectively.

### 3.2 Classification using decision trees

We generated loading matrices for first three principal components, which contained genes, ranked according to their level of contribution in generation of principal components, respectively. Top‐ranked genes with maximum contribution from loading matrices were used to classify the experimental and control samples for validation purpose using decision trees with a train test split ratio of 85 to 15, respectively. We used entropy with information gain to measure the split quality. Classification performance was evaluated using accuracy scores and confidence matrix. The accuracy score of classification model executed on validation data (15% of total data) is 0.98, whereas confidence matrix contained 86% true negatives, 100% true positives, 0% false positives or type‐I error, and 14% false negative or type‐II error. Top‐ranked loading matrix genes are given in Supporting Information S3 Table.

### 3.3 PCA DEGs

**FIGURE 2:**
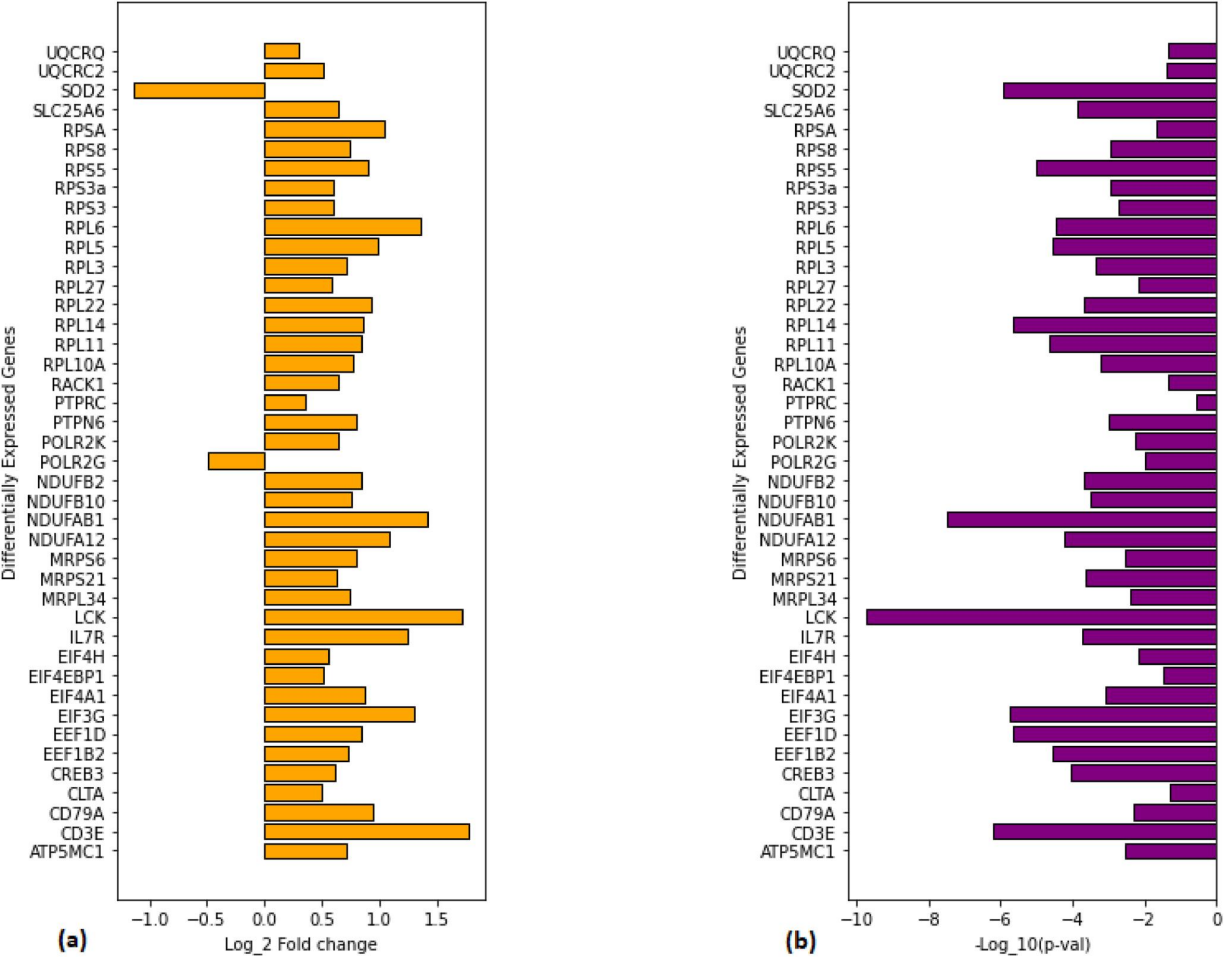
Bar charts representing differentially expressed genes from PCA. (a) Log2FC values for PCA‐enriched DEGs, (b) ‐Log10 *p*‐values for PCA‐enriched DEGs.

**FIGURE 3:**
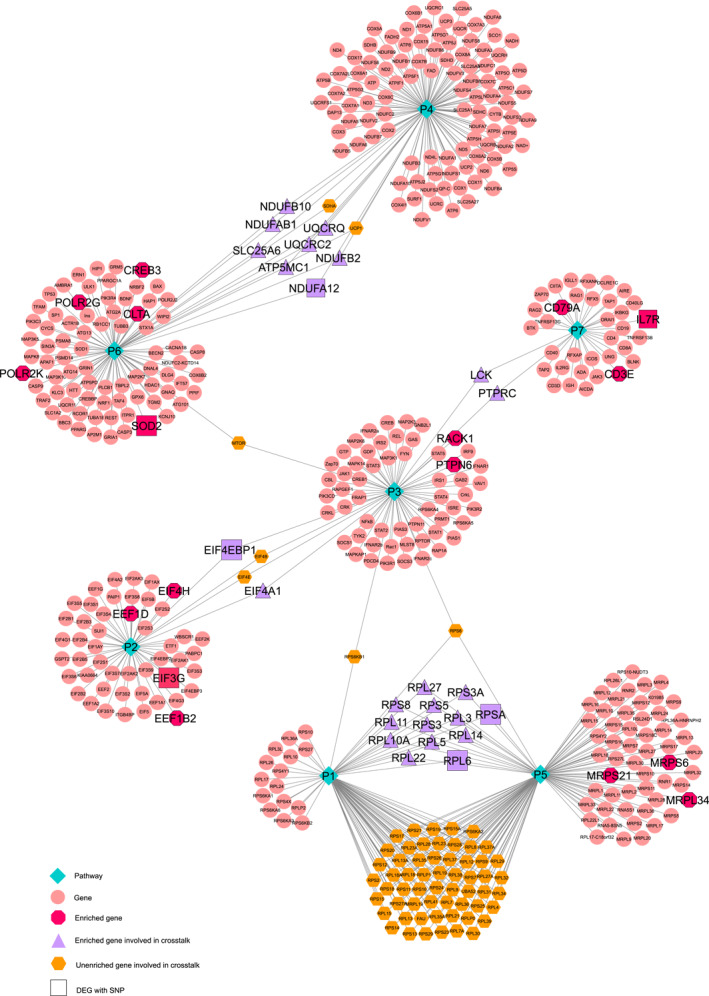
Gene‐pathway network for PCA‐enriched DEGs. Gene‐pathway network containing differentially expressed genes as genomic biomarkers for glioblastoma using PCA; their involvement in crosstalks; and their variants associated with various brain disorders. 60% of biomarkers are involved in the crosstalks between seven pathways depicted as green diamonds, that is, P1: Cytoplasmic Ribosomal Proteins, P2: Translation Factors, P3: Interferon type I signalling pathways, P4: Electron Transport Chain (OXPHOS system in mitochondria), P5: Ribosome, P6: Huntington disease, P7: Primary immunodeficiency. Pink circles represent unenriched genes, enriched genes are represented by dark pink octagons, purple triangles represent enriched crosstalking genes, whereas big squares of purple and dark pink colour illustrate the genes associated with brain disorders upon SNP analysis.

We performed enrichment analysis on top‐ranked genes followed by their validation and out of these genes, 42 were found enriched in 7 pathways; where most of the enriched genes belong to ribosomal pathways (cytoplasmic ribosomal proteins and ribosome) and some to immunodeficiency (primary immunodeficiency and interferon type I signalling), translation factors, electron transport chain, and Huntington's disease pathways. These identified pathways hold a pivotal importance in this study as they foster cancer hallmarks, which are the backbone of this study. Ribosomal pathways are responsible for playing two major roles, namely ribosomal assembly and protein synthesis. These pathways are critical for this study because ribosomal flaws at the molecular level are linked to cancer [40]. Immune system is responsible for tissue homoeostasis and protection against infectious pathogens. Tissue architecture is badly destroyed when this homoeostasis is disturbed resulting in tissue remodelling, DNA and protein alterations because of excessive oxidative stress; in turn increasing risks for cancer onset. Therefore, perturbed immune pathways end up increasing the chances for cancer development [41]. Apart from these, cancer is notorious for dysregulated translation in cells. Most of the reported cancer‐associated mutations occur in translational pathways or pathways nurturing the process of translation. mRNA translation into protein, shapes the gene expression process; therefore, tumourigenic initiation factors, and genetically altered translational machinery (defected ribosomes as mentioned above) bring in elevated risks for cancer [42]. Another infamous constituent backing up the cancer hallmarks is altered ROS (reactive oxygen species) homoeostasis. ROS are formed when escaped electrons from ETC (electron transport chain) react prematurely with oxygen molecules. Since ROS affects signalling pathways controlling cell survival and proliferation, thus, in cancer oncogenic pathways, confiscate ETC, rendering it malfunctioned to intensify ROS production [43]. Huntington's disease (HD) is a progressive neurodegenerative disorder caused by inheriting a defected huntingtin gene. Contrary to the other identified pathways, HD lowers the cancer risk [44] but we have identified crosstalks between HD and ETC pathway so it may indirectly contribute in the oncogenesis. Identified DEGs in the aforementioned pathways can be seen in Figure 2; and their crosstalks are depicted in Figure 3. However, Table 1 illustrates the enriched crosstalking DEGs alongside their general and cancer‐relevant roles.

**TABLE 1:** Identified crosstalking DEGs for glioblastoma (using PCA) with their crosstalk pathways alongside reported processes where they are involved.

Gene	Processes reported in literature	Identified pathways in this study
RPL5	Ribosomal assembly [45], tumour suppression [46, 47]	CRP, ribosome
RPL3	Ribosomal assembly [45], tat‐mediated transactivation [48], apoptosis and cell migration [49]	CRP, ribosome
RPSA	Matrix adhesion [50], tumour progression [51]	CRP, ribosome
RPS5	Translation [52], cell differentiation and apoptosis [53]	CRP, ribosome
RPL10 A	Ribosomal assembly [54], hub gene in breast cancer [55]	CRP, ribosome
RPL6	Ribosomal assembly [45], p53 stabilisation [56]	CRP, ribosome
RPS3	Encodes ribosomal protein (40S subunit), apoptosis, and DNA repair [57]	CRP, ribosome
RPL11	Ribosomal assembly [45], tumour suppression [58]	CRP, ribosome
RPL14	Ribosomal assembly [45], tumourigenesis [59]	CRP, ribosome
RPS8	Maturation of the 18S rRNA [60]	CRP, ribosome
RPS3A	Apoptosis [61], NF*κβ* signalling pathway [62]	CRP, ribosome
RPL22	Ribosomal assembly [45], tumour suppression [63]	CRP, ribosome
RPL27	Ribosomal assembly [45]	CRP, ribosome
UQCRQ	Electron transport chain [64]	ETC, HD
NDUFB10	Electron transport chain [65]	ETC, HD
NDUFA12	Electron transport chain [65]	ETC, HD
SLC25A6	ATP exchange [66], apoptosis [67]	ETC, HD
NDUFB2	Electron transport chain [65]	ETC, HD
NDUFAB1	Electron transport chain [65]	ETC, HD
UQCRC2	Electron transport chain [68], tumourigenesis [69]	ETC, HD
ATP5MC1	ATP synthesis [70]	ETC, HD
LCK	T Cell signalling [71], cell proliferation [72]	ETC, HD
PTPRC	T and B cell signalling [73], tumourigenesis [74]	ETC, HD
EIF4A1	Translation [75], metastasis [76]	ETC, HD
EIF4EBP1	Translation repression [77], tumourigenesis (phosphorylated) [78], tumour suppression (unphosphorylated) [79]	ETC, HD

Abbreviations: CRP, Cytoplasmic ribosomal proteins; ETC, Electron Transport Chain (OXPHOS system in mitochondria); HD, Huntington disease.

### 3.4 Gene set enrichment analysis

We performed Gene set enrichment analysis on the preprocessed data, which provided with 136 upregulated gene sets out of 172 in the experimental phenotype. Out of which 7 gene sets appeared significant on the basis of nominal *p*‐value, that is, <5%. Whereas no significant gene set appeared in the control phenotype. The seven gene sets mentioned correspond to pathways, which are being upregulated in several cancers, and each pathway contains some genes from our dataset other than its own genes, which have been found upregulated in these pathways with adapted pathway names (see Table 2). Supporting Information S4 Datasheet contains original pathway names as per GSEA result files. These include P53, YAP conserved, PRC2, ErbB2, STK33, and two MAPK pathways (constituting RAF and MEK regulation) comprising of 16, 8, 19, 22, 13, 18, and 20 genes from our dataset out of 45, 17, 38, 67, 39, 48, and 54 genes of their own, respectively.

**TABLE 2:** Glioblastoma genes enriched in cancer regulating pathways.

Pathway/geneset	Role	Nom‐pval	NES	Genes
RAF‐MAPK signalling pathway	Oncogenic (uncontrolled cell proliferation and Apoptosis resistance)	0.001	1.76	SNAP23, GPD2, AOPEP, SLC24A3, TFPI, PCSK6, WASF3, DKK1, SH3BGRL, DDIT4, TCN1, TSC22D3, CAV1
P53 pathway	Tumour suppressor (apoptosis and cell cycle arrest)	0.005	1.77	RNF11, LTBP1, UQCRH, S100P, CEACAM6,SDC4, IFI27, MAP3K5, ZNF185, SH3GLB1, RABGAP1L, MEST, DAAM1 DKK1, RP2, AREG,
PRC2‐EZH2 pathway (cell fate Transition)	Oncogenic (cell proliferation), tumour suppressor (inhibits cell proliferation)	0.006	1.78	KIAA0513, ST6GAL1, PKIA, DUSP3, TBXAS1, PIK3CB, SLC2A3, PDGFA, VWA5A, SLPI, ABLIM3, TNFAIP6, CLIP1, MCTP1, CLEC4E, ORAI2, MEGF9, HYAL3, IDS
YAP (Hippo signalling pathway)	Oncogenic (cell proliferation and suppression of Apoptotic genes)	0.011	1.76	THBS1, TNS1, ITGB5, SERPINE1, DAB2, FLNA, CAVIN2, FSTL1
MEK‐MAPK signalling pathway	Oncogenic (uncontrolled cell proliferation and Apoptosis resistance)	0.015	1.63	SLC6A6, GUCY1B1, LGALSL, S100P, CEACAM6, CAPN2, PKIA, FHL1, SAMD4A, ENO2, ACSL1, MEF2C, SDCBP, NME4, RAB5C, ARHGDIB, IGF2BP2, CRAT
ERBB2 signalling pathway	Oncogenic (promotes metastasis)	0.018	1.59	KIAA0513, SLC6A6, GUCY1B1, CDS2, S100P, CEACAM6, NNT, DAB2, GUCY1A1, TENT5C, FMO5, CAPN2, RAB5C, PDE4D, BNIP3L, ENO2, SDCBP, PAPSS2, NME4, GNAI2
STK33 pathway	Oncogenic (cell migration and tissue invasion)	0.037	1.51	SH3BGRL2, VCL, MTURN, CALD1, PRKAR2B, RAB27 B, BPI, MSRB3, DEFA4, HBA2, PIK3CB, MS4A3, SLPI, CAST, TENT5C, SNTB1, ABO, ABHD14 B, BICD1, CPED1, PFKM, CXCL16

*Note*: These genes can serve as genomic indicators for Glioblastoma since they are found enriched in certain cancer pathways indicating their crucial role in the onset of disease.

P53 serves as a tumour suppressor, which significantly maintains the homoeostasis of cell in stress conditions, such as hypoxia, sepsis, DNA damage, hypotension, and heat shock. Under these stimuli, p53 gets activated and actuates the cell signalling cascade which either causes apoptosis or cell cycle arrest [80].

YAP serves as a transcriptional regulator for Hippo signalling pathway. It is remarkably involved in the maintenance and development of homoeostasis at tissue level. Its activity along with TAZ plays a pivotal role in cell proliferation, tissue amplification during renewal, and regeneration and organ growth [81].

Enhancer of Zeste Homologue 2 (EZH2) is a catalytic subunit of Polycomb Repressive Complex 2 (PRC2) [82]. PRC2 is critical for cell fate transition and cell proliferation. According to functional studies made about PRC2, it acts as an oncogene in some cancers and a tumour suppressor in others by promoting and inhibiting cell proliferation, respectively. But in glioblastoma, it serves as a tumour suppressor where its one component SUZ12 actively participates in inhibiting cell proliferation [83].

ErbB2 belongs to ErbB receptor family and its receptor signalling pathway is capable of promoting metastatic activities in cancer [84].

Serine/Threonine kinase 33 (STK33) serves as an oncogene as it has been found involved in pathways regulating cell proliferation, differentiation, tissue invasion, metastasis, and tumour development [85].

MAPK pathways play crucial roles in cell proliferation, migration, differentiation, and apoptosis [86].

### 3.5 GSEA DEGs

**FIGURE 4:**
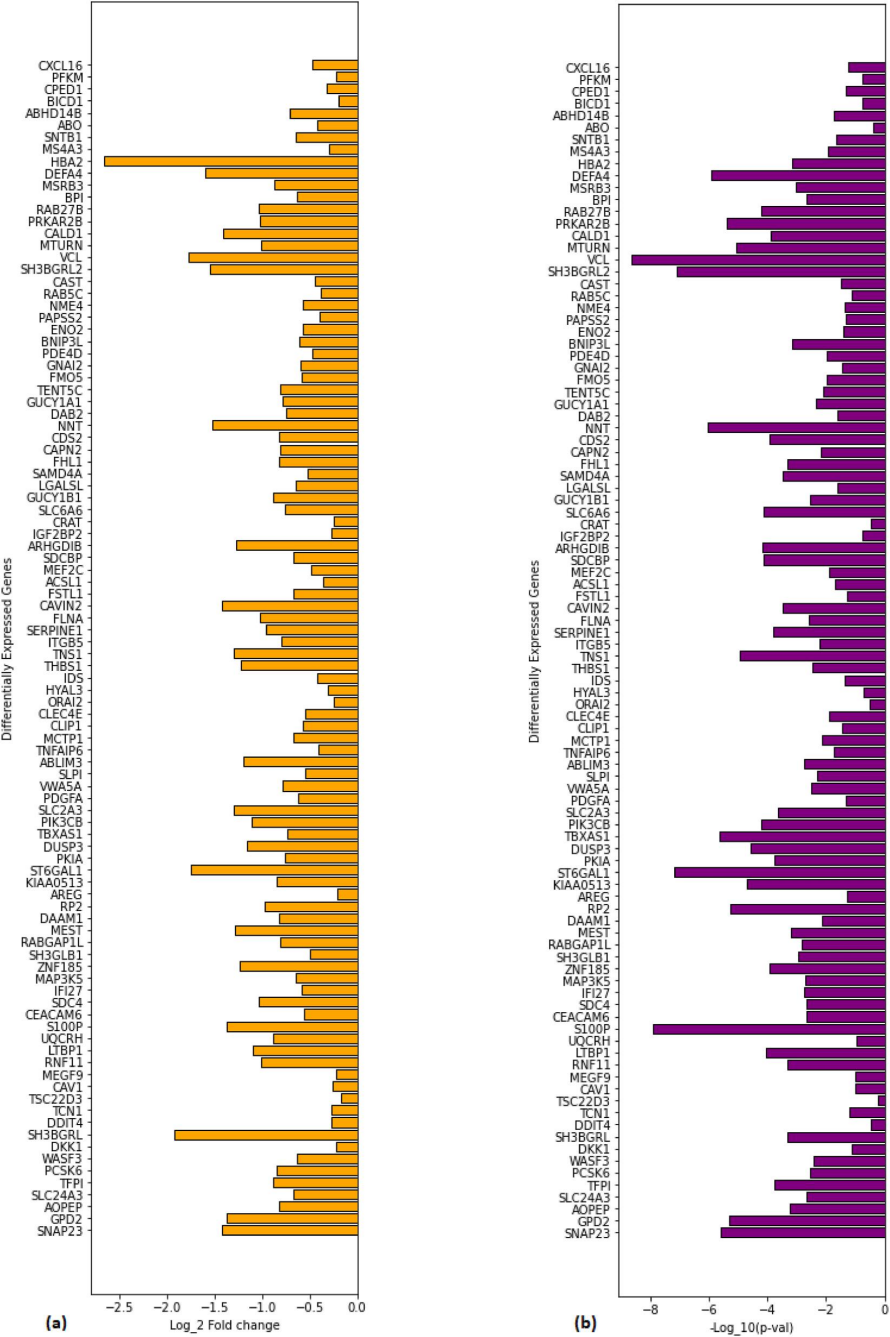
Bar charts representing differentially expressed genes from GSEA. (a) Log2FC values for GSEA enriched DEGs and (b) ‐Log10 *p*‐values for GSEA‐enriched DEGs.

**FIGURE 5:**
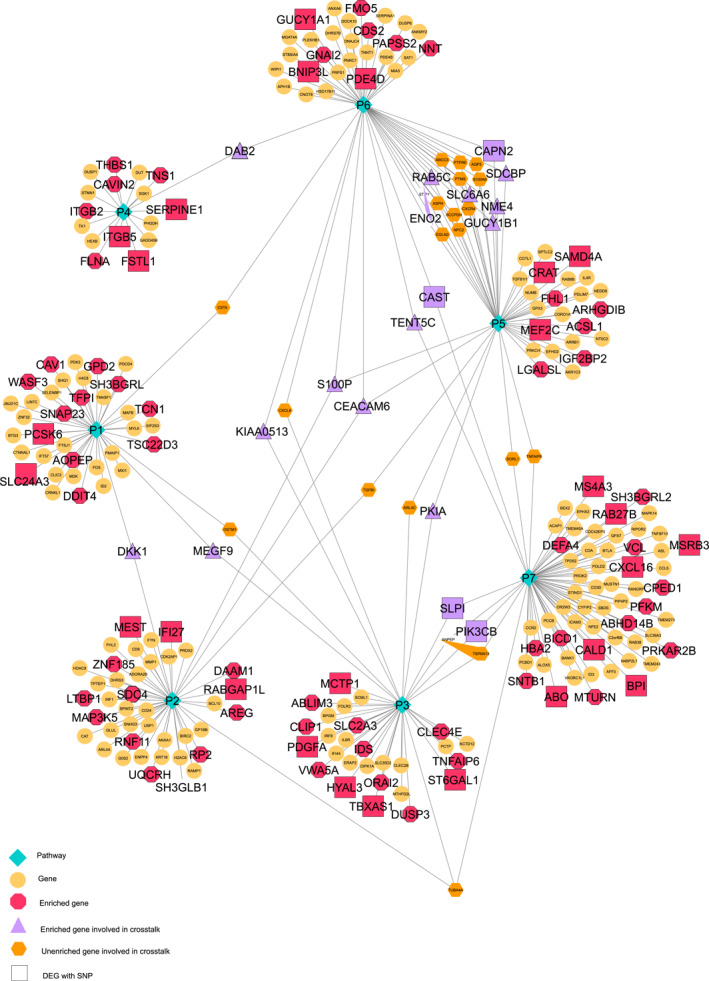
Gene‐pathway network for GSEA enriched DEGs. Gene‐pathway network containing differentially expressed genes as genomic biomarkers for glioblastoma using GSEA; their involvement in crosstalks; and their variants associated with numerous brain disorders. 18% of biomarkers are involved in the crosstalks between seven enriched oncogenic pathways, which abet cancer hallmarks depicted as green diamonds, that is, P1: RAF‐MAPK signalling pathway, P2: P53 signalling pathway, P3: PRC2‐EZH2 signalling pathway, P4: YAP‐conserved (Hippo signalling pathway), P5: MEK‐MAPK signalling pathway, P6: ERBB2 signalling pathway, P7: STK33 pathway. Yellow circles represent unenriched genes, enriched genes are represented by dark pink octagons, purple triangles represent enriched crosstalking genes, whereas big squares of purple and dark pink colour illustrate the genes associated with brain disorders upon SNP analysis.

We have identified 98 DEGs for glioblastoma using GSEA (see Figure 4) out of which 17 are actively crosstalking between enriched pathways, namely DKK1, MEGF9, S100P, CEACAM6, DAB2, SLPI, PIK3CB, PKIA, TENT5C, KIAA0513, CAPN2, SDCBP, NME4, RAB5BC, SLC6A6, ENO2, and GUCY1B1, which are quite evident in Figure 5.

### 3.6 Single‐nucleotide polymorphism (SNP) analysis

We performed SNP analysis on the set of DEGs we identified using PCA and GSEA, which comprises a total of 139 genes, 42 from former and 97 from later. All of these genes are found associated with cancer fostering pathways in this study. This analysis is performed to better understand the onset of Glioblastoma as SNPs help to understand the aetiology of diseases. In Supporting Information S5 Datasheet and S6 Datasheet, we have mentioned the queried genes in DisGeNET involved in brain disorders, alongside their SNPs reporting highest DisGeNET disease association score. A total of 36 DEGs (6 from PCA and 30 from GSEA) have been identified from this analysis having one or more SNPs associated with brain disorders (see Table 3 and Table 4).

**TABLE 3:** SNPs association with brain disorders for PCA DEGs.

Gene	SNPs	Disease(s) associated with SNP
EIF3G	rs3826784	Narcolepsy, Narcolepsy 1
	rs2305795	Narcolepsy, Narcolepsy 1
EIF4EBP1	rs1208512188	Anxiety disorders
	rs1276624859	Bipolar disorders, anxiety disorders, anxiety
NDUFA12	rs249153	Alzheimer's disease
	rs249166	Alzheimer's disease
	rs249167	Alzheimer's disease
RPL6	rs1214031315	Pilocytic astrocytoma, childhood pilocytic astrocytoma, adult pilocytic astrocytoma
RPSA	rs747760223	Alzheimer's disease, Parkinson's disease
SOD2	rs4880	Parkinson's disease, epilepsy
	rs953038635	Schizophrenia, Parkinson's disease, major depressive disorder, Alzheimer's disease

**TABLE 4:** SNPs association with brain disorders for GSEA DEGs.

Gene	SNPs	Disease(s) associated with SNP
ABO	rs505922	Ischaemic stroke
	rs529565	Ischaemic stroke, cerebrovascular accident
BNIP3L	rs73219805	Schizophrenia
	rs1042992	Schizophrenia
	rs77609452	Impaired cognition
BPI	rs6024905	Psychotic disorders, mental disorders
CALD1	rs756573441	Huntington's disease
CAPN2	rs760073870	Tauopathies
CAST	rs1235134025	Tauopathies
	rs1559085	Sporadic Parkinson's disease
	rs752410089	Tauopathies
CRAT	rs138665095	Progressive neurologic deterioration
	rs141970897	Leigh disease
	rs762425351	Leigh disease
CXCL16	rs2277680	Cerebrovascular accident
FSTL1	rs1700	Schizophrenia
GUCY1A1	rs7692387	Cerebrovascular accident
HYAL3	rs1377666593	Parkinson's disease
IFI27	rs544899118	Squamous cell carcinoma of the head and neck
ITGB5	rs7373878	Major depressive disorder
MCTP1	rs17418283	Bipolar disorder
MEF2C	rs1554139771	Movement disorders
	rs1065861	Schizophrenia
	rs1561824498	Autism spectrum disorders
MEST	rs863223353	Schizophrenia, childhood
MS4A3	rs474951	Alzheimer's disease
MSRB3	rs61921502	Alzheimer's disease, cerebrovascular accident
PCSK6	rs11855415	Dyslexia
PDE4D	rs7732249	Schizophrenia
	rs966221	Ischaemic stroke
PDGFA	rs755794544	Adult glioblastoma, childhood glioblastoma, glioblastoma, glioblastoma multiforme
PIK3CB	rs752021744	Tumour progression
RAB27 B	rs1833288	Major depressive disorder
RABGAP1L	rs17301853	Common migraine, migraine disorders
	rs75650221	Major depressive disorder
SAMD4A	rs4901536	Schizophrenia
SLC24A3	rs3790171	Neuroblastoma
	rs4814864	Migraine disorders
	rs6081613	Migraine disorders
SLPI	rs771884087	Nervous system disorder
SERPINE1	rs763351020	Ischaemic stroke
	rs1349041080	Severe dementia
	rs1442033697	Tumour cell invasion
	rs1799768	Epilepsy, temporal lobe
	rs1799889	Meningitis, pneumococcal
ST6GAL1	rs3936289	Alzheimer's disease
TBXAS1	rs10277664	Schizophrenia
	rs10487667	Ischaemic stroke
	rs41708	Cerebrovascular accident

## 4 DISCUSSION

Glioblastoma exists as one of the deadliest cancers in the history of mankind till date; because when it is diagnosed, the maximum of the damage has been done already leaving its sufferers with a small life expectancy rate [5]. Since it is intracranial cancer, surgery coupled with chemo and radiotherapy is the only resort [4]. Cancer usually occurs as a consequence of genomic instability; therefore, genomic biomarkers always play a pivotal role in order to understand the dynamics, diagnosis, prognosis, and treatment of disease [87]. In this study, we have used TEP data (which comes from liquid biopsies that are non‐invasive in nature and can be obtained at any time over the course of disease) to identify genomic biomarkers for glioblastoma. The obtained results hold quite significant information in order to understand the onset and prevalence of disease.

In this study, we have identified genomic biomarkers for glioblastoma using principal component analysis [28] as a non‐canonical and gene set enrichment analysis [36] as a canonical method. The former one is used with SVD (singular value decomposition) as a dimensionality reduction approach on the RNA‐seq dataset of glioblastoma obtained from ArrayExpress containing 39 experimental and 43 control samples. The high‐dimensional RNA‐seq data has been reduced to smaller dimensions and volume in order to preserve significant gene signatures by reducing multicollinearity to represent the rest of the data. Reduced signatures (aka features) obtained from the first three PCs were further used by the decision tree algorithm to classify healthy and control samples to check for their representation (predictive) potential, which was evaluated with an accuracy score of 0.98. Whereas the latter approach is used on the same dataset to interpret gene expression by focusing on the group of genes either performing similar biological functions, regulating the same pathway, or sharing a common location on the chromosome. Both approaches provided different results, which highlight the fact that different methods come out with different results that being noteworthy help in solving the mega‐puzzle of disease dynamics by providing a sensible understanding.

**FIGURE 6:**
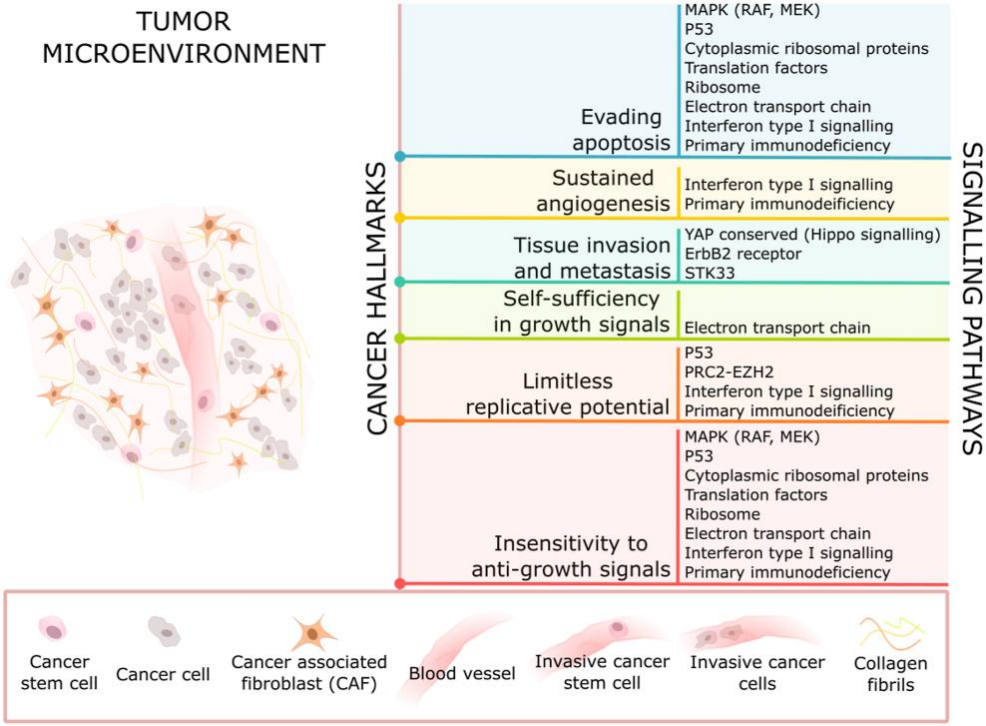
Cancer hallmarks. Enriched signalling pathways from GSEA and PCA alongside their cross‐connection with cancer hallmarks.

The DEGs from PCA appear enriched in seven pathways responsible for protein translation (as it involves ribosomal pathways), electron transport chain, immunity, and Huntington's disease, which indirectly foster some cancer hallmarks (see Figure 6). A big proportion of enriched pathways correspond to ribosomal functions, which play a vital role in translation from mRNA to protein. Defects in ribosomes have been linked to cancer or ribosomopathies. Ribosomal mutations can either be congenital or somatic, the former results in ribosomopathies, which increase the risk of developing cancer whereas, the latter results in various cancers. Since ribosomes serve as machinery for protein translation; therefore, defective ribosomes hardly yield functional protein poly peptides [40]. The enriched and crosstalk genes in ribosomal pathways are RPL3, RPL5, RPL6, RPL10 A, RPL11, RPL14, RPL22, RPL27, RPSA, RPS3A, RPS3, RPS5, and RPS8. The RPLs belong to large and RPSs belong to a small subunit of ribosome [88] and out of large subunit proteins, RPL5, RPL10, and RPL11 appear quite famous in literature for undergoing somatic mutations [40]. RPL3, RPL5, RPL6, RPL10 A, RPL11, RPL14, RPL22, RPL27, RPS3, and RPS8 are crucial for ribosome assembly [45, 54], whereas RPSA, RPS3A, and RPS5 play their role in matrix adhesion [50], NF*κβ* signalling pathway [62], and translation [52], respectively. Alterations in these genes have been found associated with several cancers as they play crucial roles in cell cycle activities also. RPL3 helps in apoptosis and cell migration [49], RPL5, RPL11 and RPL22 in tumour suppression [46, 47, 58, 63], RPL6 in p53 stabilisation [56], RPL10 A appears as a hub gene in breast cancer [55], RPL14 in tumourigenesis [59], RPSA in tumour progression [51], RPS3A in apoptosis [61], RPS3 in apoptosis and DNA repair [57], while RPS5 in cell differentiation and apoptosis [53]. Moreover, apart from regulating translation, some ribosomal genes also regulate certain genes like MDM2, which inhibit p53. Under conditions like starvation, environmental stress (intra or inter‐cellular), or the presence of anti‐growth signals, ribosome biogenesis is stalled, which results in the freedom of ribosomal proteins from MDM2 complexes resulting in p53 activation. P53 under such grueling conditions induces cell cycle arrest and apoptosis. Normal or low levels of these proteins are important for p53 regulation but increased levels cause problems, deviating the system from normal behaviour to oncogenic behaviour [40] where tumour cells replicate rapidly avoiding the anti‐growth and apoptotic signals.

Reactive oxygen species (ROS) are capable of controlling signal transduction pathways, and on the basis of their levels, can either be oncogenic or tumour suppressive. There are several sources of ROS generation in the cell, that is, mitochondria, endoplasmic reticulum, and peroxisomes (high oxygen consumption), which produce ROS in response to cytokines, foreign particle invasion, xenobiotics etc. A major source for ROS production is the electron transport chain (ETC) in mitochondria aka the mitochondrial respiratory chain. ROS are formed when electrons escape from ETC and react with oxygen molecules, and their perturbed levels can affect cell survival and proliferation. However, some oncogenic pathways confiscate ETC to increase ROS production in order to keep tumour phenotype [43]. In our study, we have identified eight enriched genes that are crosstalking between mitochondrial ETC and Huntington's disease (HD) pathway. These common DEGs include ATP5MC1, NDUFA12, NDUFAB1, NDUFB10, NDUFB2, SLC25A6, UQCRC2, and UQCRQ. ATP5MC1 and SLC25A6 are responsible for ATP synthesis [70] and ATP exchange [66], respectively; NDUFA12, NDUFAB1, NDUFB10, and NDUFB2 encode proteins, which are sub‐units of NADH dehydrogenase, which is one of the five complexes of electron transport chain [65], whereas UQCRC2 and UQCRQ participate in electron transport chain [64, 69]. HD is a progressive neurodegenerative disorder that causes hyperkinetic musculature problems as certain parts of the brain get damaged over time, which is responsible for muscle coordination and the development of motor skills. It is a hereditary disease caused by inheriting a defective huntingtin gene (HTT) from at least one of the affected parents. Surprisingly, HD reduces the risk of developing cancer in its patients [44]. Though HD pathway does not foster any of the cancer hallmark pathways as mentioned above, some of the DEGs that appeared enriched in them are responsible for crosstalk between HD and ETC pathways; which may indirectly contribute to tumour progression. Thus, from these genes, only SLC25A6 and UQCRC2 participate in apoptosis [67] and tumourigenesis [69], respectively.

Cancer always puts the immune system of the body at stake. Immune cells are responsible for killing the diseased and infected cells in order to maintain a healthy environment inside the body, but unfortunately, tumour cells evade the immune system causing havoc to normal health [41]. In this study, we have identified four enriched crosstalk genes in primary immunodeficiency, Interferon type I signalling, and translation factors pathways; that is, LCK, PTPRC, EIF4A1, and EIF4EBP1. The former two genes are crosstalking between the first two pathways and the latter two are crosstalking between the latter two pathways, respectively. People suffering from PI have malfunctioned immune system and therefore, they are more prone to get infected and become grievously sick. Moreover, they are at higher risk of developing cancers [89]. Whereas interferon signalling is one of the critical pathways in human immune response where it increases cellular resistance and ceases cell growth against viral infections. Immune dysfunction appears as a consequence of cancer affecting different immune pathways. In experimental groups, interferon type I signalling has been found reduced in adaptive immune cells (T and B) as compared to control healthy groups due to certain alterations in the involved proteins [90]. Immuno compensation renders the tumour cells to evade apoptosis by bypassing all the anti‐growth signals and proliferate in an uncontrolled fashion with sustained angiogenesis [41]. The general function of LCK and PTPRC is T and B cell signalling [71, 73] but regarding cancer, they participate in cell proliferation [72] and tumourigenesis [74], respectively. Whereas EIF4A1 and EIF4EBP1 are responsible for translation [75] and translation repression [77], respectively; but with respect to cancer EIF4A1 promotes metastasis [76], while EIF4EBP1 promotes tumourigenesis when phosphorylated [78] and suppresses tumourigenesis when unphosphorylated [79].

However, the DEGs we obtained from GSEA appear enriched in pathways that foster several cancer hallmarks (see Figure 6). As mentioned in the previous section, GSEA results include MAPK (Raf, MEK) [86], P53 [80], PRC2‐EZH2 [82, 83], YAP conserved (Hippo signalling) [81], ErbB2 [84], and STK33 [85] signalling pathways. The MAPK pathway is critical to cell survival and growth. Mutations in these pathways result in uncontrolled cellular proliferation and apoptotic resistance where cell death signals are bypassed. Its main signalling molecules include ERK, MEK, Ras, and Raf. Raf and MEK belong to the ERK module (pathway), which is one of the most studied MAPK pathway, where MEK phosphorylates ERK and itself gets phosphorylated by Raf. Dysregulations in this module of MAPK often make it a culprit for tumourigenesis [86]. The crosstalking enriched genes in the Raf‐MAPK pathway are DKK1 and MEGF9; former crosstalks with P53 signalling pathway, whereas later crosstalks with PRC2‐EZH2 pathway. Generally, DKK1 participates in embryonic development [91] but in cancer, it regulates tumour progression as it is a well‐known inhibitor of Wnt signalling pathway [92]. MEGF9 is mainly expressed in the brain where its translated protein acts as a signalling molecule [93].

P53 is able to maintain genomic integrity in the body by activating repair mechanisms if any damage in DNA has been encountered. It either fixes the damage and cell functions normally or if the damage is irreparable, then kills the cell by initiating apoptosis or halts the cell by inducing cell cycle arrest. This whole process involves a p53 transduction pathway where the levels of p53 are high. Its loss of function has been observed in most cancers where cells bypass all apoptotic signals and keep proliferating [94]. Apart from DKK1, we have identified S100P and CEACAM6 as enriched crosstalking DEGs where the former cross communicates between P53 signalling pathway and MEK‐MAPK signalling pathway and later does between P53 signalling pathway and ERBB2 signalling pathways. S100P participates in cell proliferation and cell migration [95]. CEACAM6 is crucial to cell proliferation and cell adhesion [96].

Proteins from the Polycomb group (PcG) appear as epigenetic factors and dysregulation in their pathways occurs as a frequent process in cancer. PRC2 comprises two catalytic sub‐units namely EZH1 and EZH2, which are responsible for its histone methyl transferase activity. Mutations in catalytic sub‐units of PRC2 have shown a positive correlation with certain cancer types. PRC2 shows both oncogenic and tumour‐suppressive roles; when EZH2 undergoes somatic mutations, its catalytic activity is hyperactivated rendering PRC2 to reveal its oncogenic side. Contrary to this mutation causing loss of function in PRC2 catalytic substrate as H3K27 turns on PRC2's tumour suppressive side [83]. This pathway has five crosstalking enriched genes, namely MEGF9, SLPI, PIK3CB, PKIA, and KIAA0513. MEGF9 is responsible for crosstalking between RAF‐MAPK and PRC2‐EZH2 pathways, SLPI and PIK3CB between PRC2‐EZH2 and STK33 pathways, PKIA between PRC2‐EZH2 and MEK‐MAPK pathways, whereas KIAA0513 between PRC2‐EZH2 and ErbB2 pathways. SLPI promotes cell proliferation and inhibits inflammation [97], PIK3CB is critical for cell growth and invasion [98] and KIAA0513 is expressed in brain tissues where it plays its role in apoptosis, cytoskeletal regulation, and neuroplasticity [99].

Hippo signalling pathway is crucial to tumourigenesis and genetic alterations in its major constituent components, that is, YAP, TAZ, MST (1, 2), and LATS (1, 2) stimulate cellular migration, neighbouring or distant tissue invasion, and malignancy of tumour cells. YAP serves as an efficient and active transcriptional regulator in tumour malignancies. Along with its paralog TAZ, it is vital for cancer initiation or tumour growth promotion. Hippo pathway with LATS activation suppresses the YAP/TAZ activity; thus, the conservation of YAP is necessary for tumourigenesis. The YAP conserved pathway serves as one of the major cancer‐promoting pathways by initiating tumour development [100]. In this pathway, we have one enriched crosstalking gene, which links it to the ErbB2 pathway, namely DAB2. It is an endocytic adaptor that is responsible for inhibiting cell growth [101].

STK33 carries a kinase domain and comes from Ca2+ calmodulin‐dependent kinase family. It shows a limited expression in most of the human tissues except for testicular cells, where its expression is very high. It has been found to have a synthetic lethal interaction with the mutated KRAS gene in several tumour cells. STK33 promotes tumourigenesis by stimulating cell migration, invasion of neighbouring tissues, and epithelial‐mesenchymal transition (EMT) [85]. This pathway constitutes of one enriched crosstalking gene with the ErbB2 pathway, that is, TENT5C. It has a close association with B cells and it has been found in the literature that it stimulates humoral immune response [102].

ErbB2 receptor initiates its pathway to assist cells in their growth, differentiation, and programmed death. The overexpression of the ErbB2 receptor turns its oncogenic switch on, making it pivotal in many human malignancies. In its overexpressed form, it enhances the metastatic potential of tumour cells [84]. We have identified seven enriched genes that are participating in crosstalks between ErbB2 and MEK‐MAPK pathways, namely CAPN2, ENO2, GUCY1B1, NME4, RAB5C, SDCBP, and SLC6A6.

Therefore, using PCA we have identified 42 DEGs, out of which RPS3A, RPL27, UQCRQ, NDUFA12, SLC25A6, NDUFB2, and ATP5MC1 have not been associated with GBM before. The diagnostic ability of these genes has been validated in Section 3.2. Moreover, using GSEA, we have identified 98 DEGs where two of these appear as new biomarkers, namely SNAP23 and UQCRH. These results had been verified using Harmonizome [103]. Their functions have been discussed above in detail, which indicate that these can be used as prognostic biomarkers for GBM.

Furthermore, SNP analysis performed on identified DEGs aided to elucidate the aetiology of glioblastoma. The variants of certain identified DEGs have associations with several brain disorders and tumours (Tables 3 and 4). These include Narcolepsy, anxiety, and bipolar disorders, Alzheimer's disease, Parkinson's disease, Huntington's disease, epilepsy, Leigh disease, schizophrenia, mental, psychotic, and major depressive disorders, Ischaemic stroke, cerebrovascular accident, impaired cognition, tauopathies, progressive neurologic deterioration, dyslexia, autism, migraine, dementia, pneumococcal meningitis, squamous cell carcinoma of head and neck, astrocytoma, neuroblastoma, and glioblastoma. This information pinpoints that the identified biomarkers in this study hold a very pivotal role in glioblastoma onset and progression as variations in their genomic sequences have already been reported to be associated with many nervous dysfunctionalities.

Biological systems are dynamical in nature and perturbations in a pathway can be viewed as a consequence of alterations in a few genes. The involvement of such genes in crosstalks between other pathways provides a wholesome view for understanding the dynamical behaviour of a system. It helps in tracing back the causes to formulate better propositions for study and finding good therapeutic targets. Most of the identified DEGs exist in literature already as probable biomarkers for glioblastoma but this study highlights their involvement in crosstalks with other pathways nurturing cancer hallmarks (either directly or indirectly) also.

## 5 CONCLUSION

This study focuses on the identification of DEGs as genomic biomarkers for glioblastoma using canonical and machine‐learning approaches, namely GSEA and PCA, respectively, and highlighting the role of these DEGs in crosstalks between pathways nurturing cancer hallmarks. The major limitation of this study is the sparse availability of data as glioblastoma is an aggressive brain tumour, and owing to its intracranial nature, it is hard to get more samples as surgeries are not excessively performed in this case. Therefore, we have used TEP data for this purpose to see how much meaningful information can be obtained. In this regard, the identified DEGs have been found enriched in important oncogenic, neurodegenerative disease, and translational pathways, which play a direct role in the onset of disease. Moreover, the identified SNPs of these biomarkers associated with numerous brain disorders enhance our knowledge regarding possible implication of these genes in disease mechanism. This work suggests that glioblastoma is regulated by complex gene interactions in several pathways simultaneously, which should be kept under consideration while proposing new therapeutic targets. These findings may contribute to comprehend the disease dynamics of glioblastoma better at molecular and genomic levels while using TEP data. This can serve as an appealing future direction for such biomarker‐based therapies using liquid biopsies.

## AUTHOR CONTRIBUTION

**Darrak Quddusi**: Conceptualisation, Data curation, Formal analysis, Investigation, Methodology, Project administration, Software, Validation, Visualisation, Writing – original draft, Writing – review and editing. **Naim Bajcinca**: Conceptualisation, Resources, Supervision, Writing – review and editing

## CONFLICT OF INTEREST STATEMENT

There is no conflict of interest.

## Supporting information

Supporting Information S1

Supporting Information S2

Supporting Information S3

Supporting Information S4

Supporting Information S5

Supporting Information S6

Supporting Information S7

## Data Availability

The data that support the findings of this study are available in Array Express at https://www.ebi.ac.uk/arrayexpress/, reference number GSE68086. These data were derived from the following resources available in the public domain: https://www.ebi.ac.uk/arrayexpress/experiments/E‐GEOD‐68086/?query=GSE68086.
