# Supplementary material for: Identification of genomic biomarkers and their pathway crosstalks for deciphering mechanistic links in glioblastoma
Source: IET Syst Biol. 2023 Jun 5;17(4):143–61. doi: 10.1049/syb2.12066 (PMC10439498; doi:10.1049/syb2.12066)
Supplement: Supplementary file 3 — Supporting Information S3 [file SYB2-17-143-s004.pdf]

The selection of a machine learning method for a problem depends on specific characteristics of the dataset (size, complexity, nature, distribution, type of variables, and features), nature of the problem being addressed, available computation resources, and desired level of interpretability [1]. This is clearly depicted in the accuracy scores and confusion matrices given below. Table 1 contains accuracy scores of six machine learning classifiers i.e. bernoulli naive bayes [2], gaussian naive bayes [2], k-nearest neighbor [3], support vector machine [4], logistic regression [5], and decision tree [6]; using 4 different gene expression datasets and the performance of each method varies with respect to each dataset. Secondly, the confusion matrix provides very critical information in terms of type I and type II errors. Type I error refers to false positives, where the predicted outcome is classified as if the underlying condition exists when in reality it does not. Contrary to this, type II error refers to false negatives, where the predicted outcome is classified as if the underlying condition does not exist when in actuality it does, rendering type II error more crucial than type I. Therefore, while selecting a classifier, apart from the accuracy score type II error in the confusion matrix is very important as well.

Dataset 1 has been used for training decision tree in the manuscript and in Table 1 the accuracy score for decision tree and SVM is greater as compared to other models which is otherwise different in case of other datasets. Also, the confusion matrices for both methods show zero type I error. Since, dataset 1 is the real chunk of data to be used in the study we picked decision tree as it outperformed other methods except SVM. Decision trees are better interpretable and manage colinearity better and efficiently than SVM.

Table 1. Machine learning methods for classification with accuracy scores on different datasets

Method	Data 1 score	Data 2 score	Data 3 score	Data 4 score
BernoulliNB	0.69	0.92	0.69	0.85
GaussianNB	0.77	0.54	0.77	0.54
KNN	0.76	0.62	0.78	0.69
SVM	0.84	0.54	0.77	0.85
Logistic Regression	0.77	0.92	0.62	0.92
Decision tree	0.84	0.77	0.77	0.92

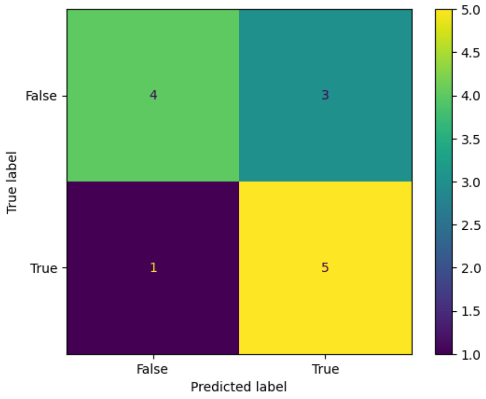

(a) Dataset 1

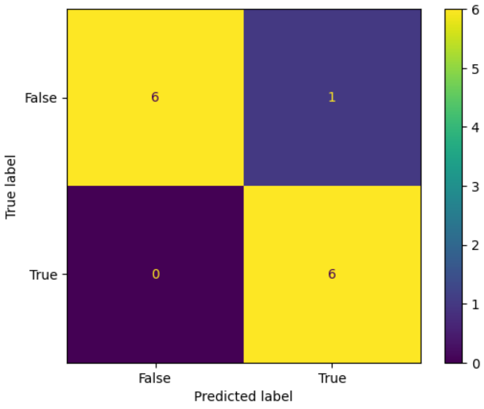

(b) Dataset 2

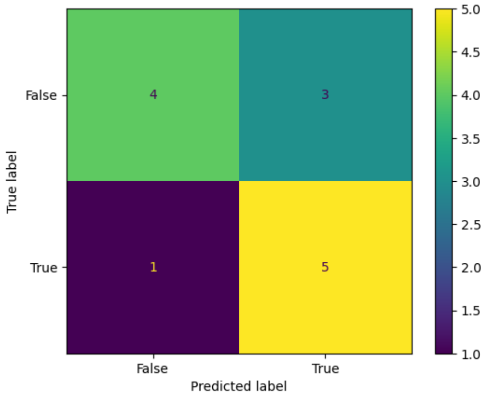

(c) Dataset 3

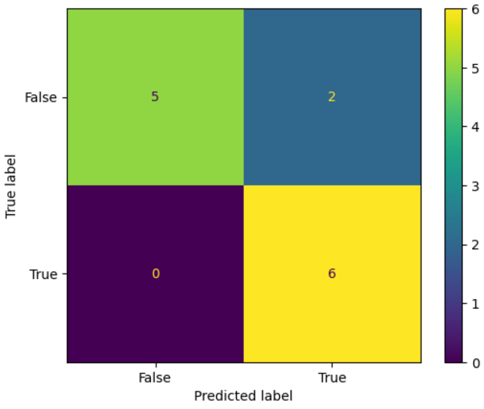

(d) Dataset 4

Fig 1. Confusion matrices for method bernoulli naive bayes classifier with different datasets.

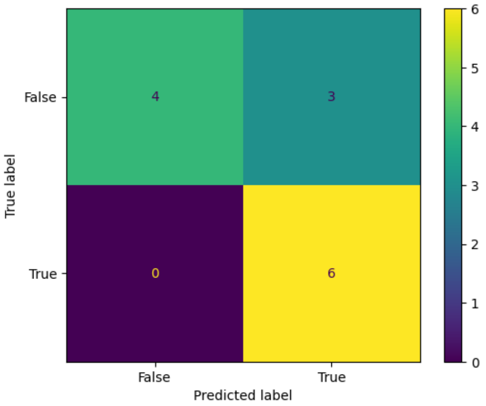

(a) Dataset 1

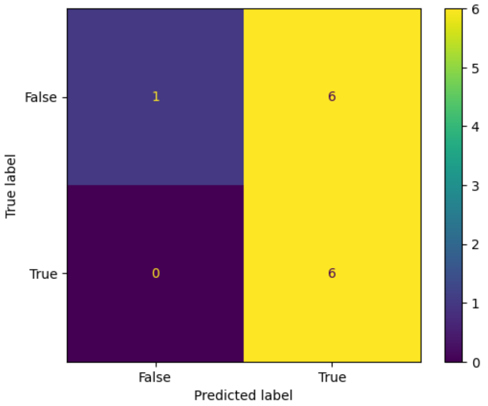

(b) Dataset 2

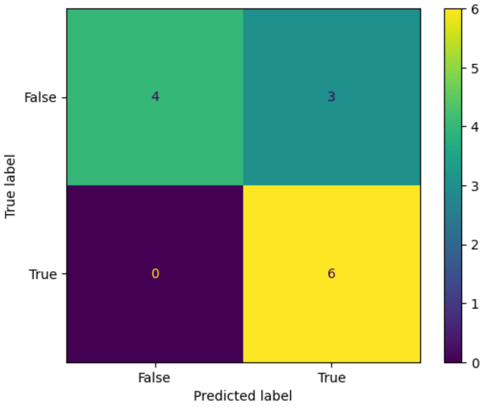

(c) Dataset 3

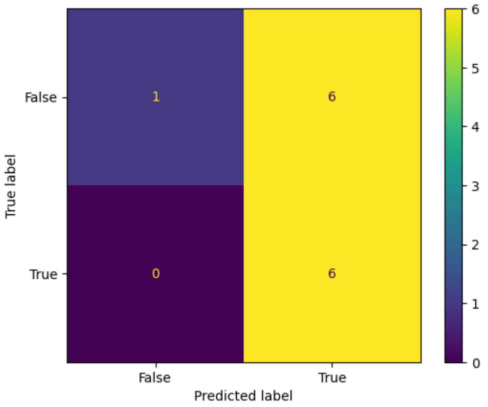

(d) Dataset 4

Fig 2. Confusion matrices for method gaussian naive bayes classifier with different datasets.

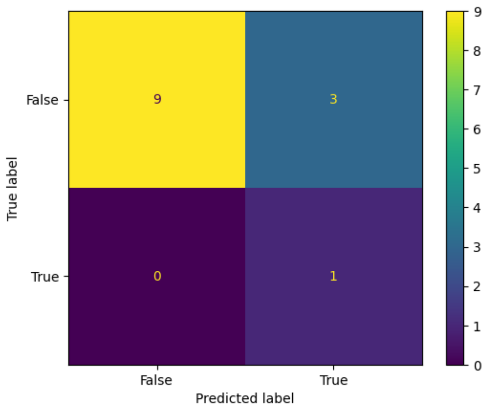

(a) Dataset 1

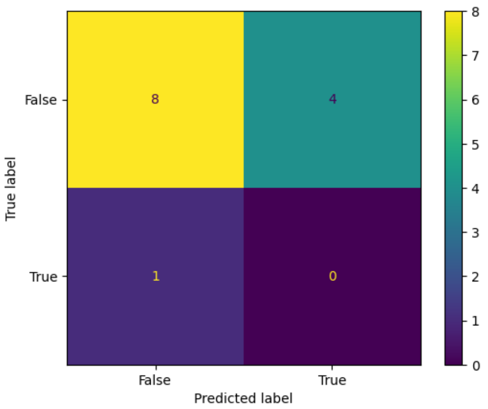

(b) Dataset 2

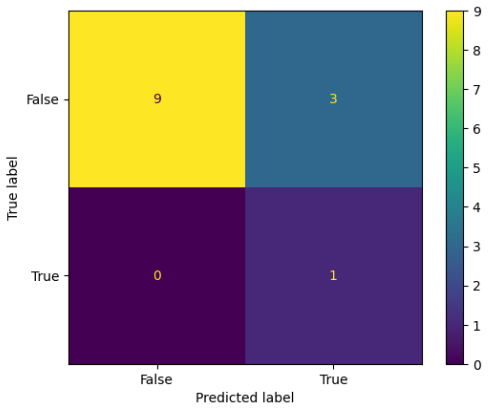

(c) Dataset 3

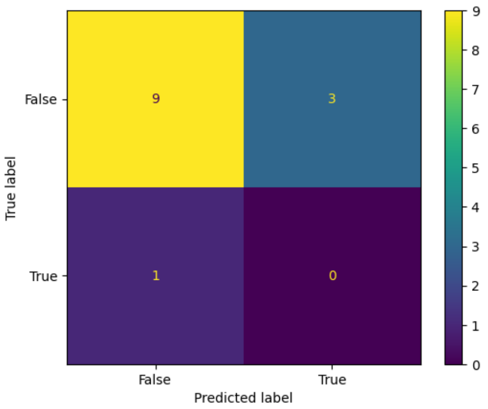

(d) Dataset 4

Fig 3. Confusion matrices for method K-nearest neighbor with different datasets.

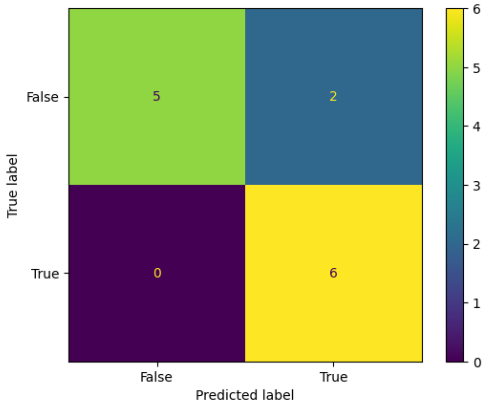

(a) Dataset 1

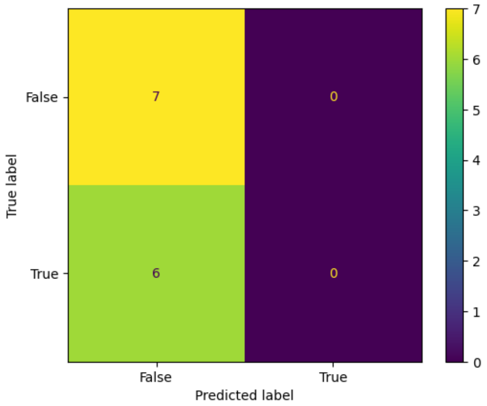

(b) Dataset 2

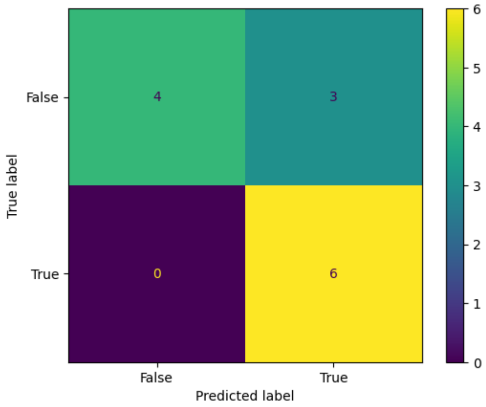

(c) Dataset 3

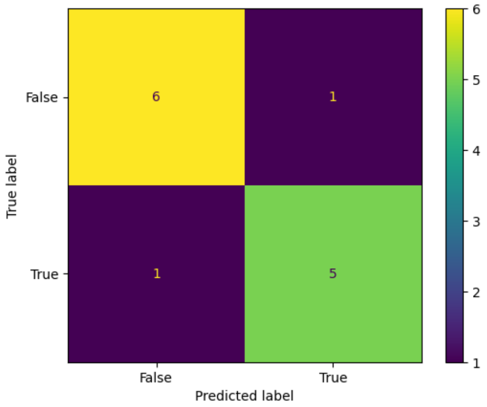

(d) Dataset 4

Fig 4. Confusion matrices for method support vector machine with different datasets.

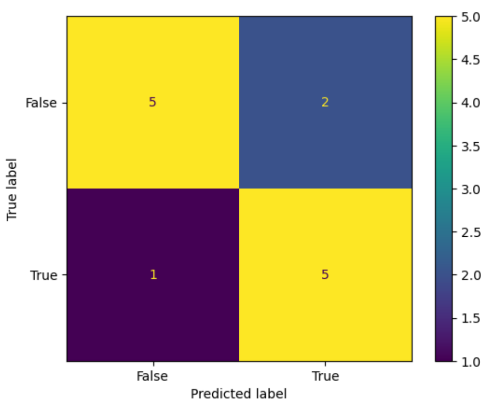

(a) Dataset 1

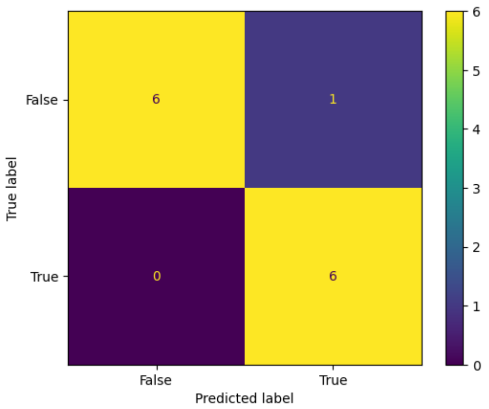

(b) Dataset 2

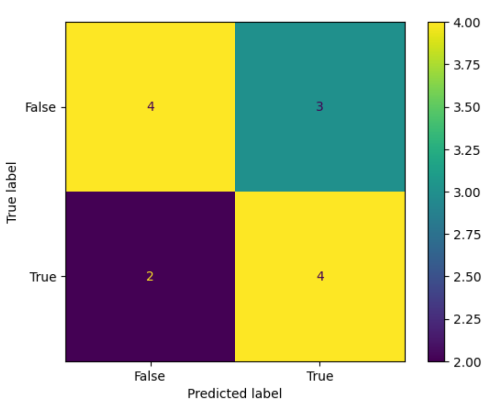

(c) Dataset 3

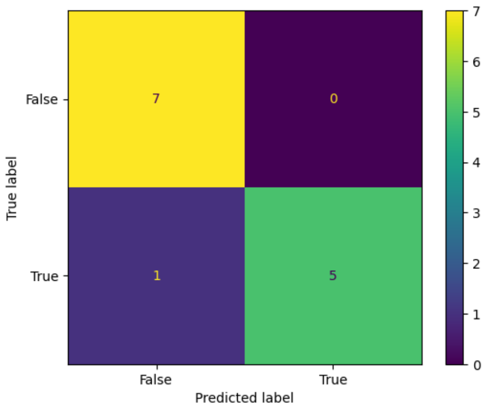

(d) Dataset 4

Fig 5. Confusion matrices for method logistic regression with different datasets.

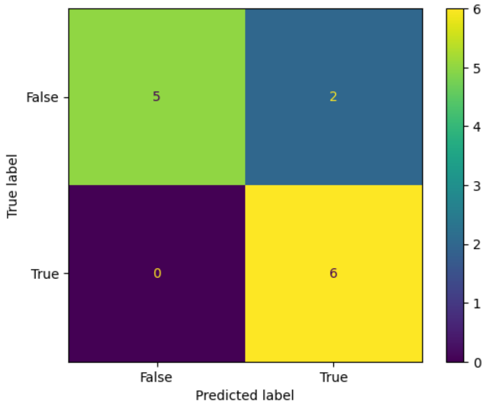

(a) Dataset 1

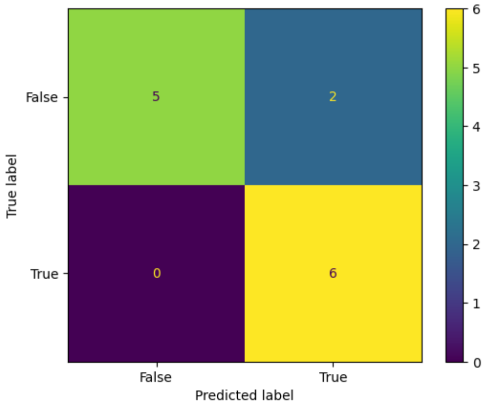

(b) Dataset 2

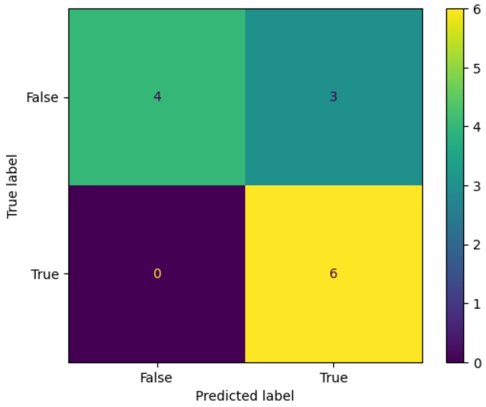

(c) Dataset 3

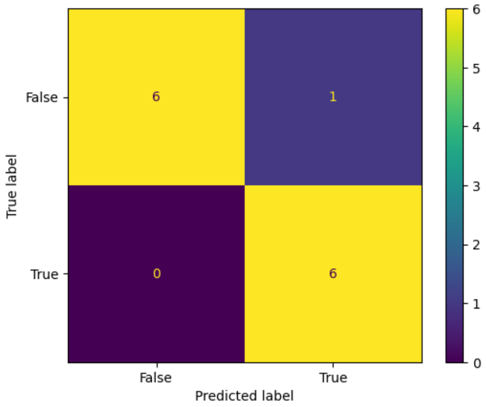

(d) Dataset 4

Fig 6. Confusion matrices for method decision tree with different datasets.

References

1. Alpaydin E. Introduction to machine learning. MIT press; 2020.
2. Singh G, Kumar B, Gaur L, Tyagi A. Comparison between multinomial and Bernoulli naïve Bayes for text classification. In: 2019 International Conference on Automation, Computational and Technology Management (ICACTM). IEEE; 2019. p. 593–596.
3. Larose DT, Larose CD. k-nearest neighbor algorithm. Wiley Data and Cybersecurity; 2014.
4. Suthaharan S, Suthaharan S. Support vector machine. Machine learning models and algorithms for big data classification: thinking with examples for effective learning. 2016; p. 207–235.
5. LaValley MP. Logistic regression. Circulation. 2008;117(18):2395–2399.
6. Rokach L, Maimon O. Decision trees. In: Data mining and knowledge discovery handbook. Springer; 2005. p. 165–192.
