# Supplementary material for: Identification of genomic biomarkers and their pathway crosstalks for deciphering mechanistic links in glioblastoma
Source: IET Syst Biol. 2023 Jun 5;17(4):143–61. doi: 10.1049/syb2.12066 (PMC10439498; doi:10.1049/syb2.12066)
Supplement: Supplementary file 4 — Supporting Information S4 [file SYB2-17-143-s006.pdf]

Table 1. Loading genes for first three PCs.

PCs	Loading genes
PC1	HNRNPA1P7, HSP90AB1, EIF3L, HNRNPA1P10, RNF114, HNRNPA1P48, SF3A3, CCT5, SARS1, EEF1A1P19, PRPF38A, EIF2A, EIF2S1, SNX2, PDHA1, EIF3C, SF3B2, CYTIP, CD53, DNAJA1, GDI2,ITGA4, EIF3D, SDHD, GIMAP2, RPL29P11, RPS5, EEF1A1P4, EEF1A1P6, RAB18, XRCC5, DDOST, USP3, GIMAP4, DKC1, CCT6A, TMED10, RAE1, EEF1A1P25, RPL21P39, ASNSD1, PPP2R1A, HSPA9, SAMHD1, PNRC2, NOB1, MAT2B, SRP54, UBE2N, LEPROTL1, SELPLG, GNL2, UQCRC2, NONO, APMAP, PTPN6, GBP1, DDX47, TES, SLC25A6, EIF3A, SMU1, SASH3, LCK, POLDIP3, FDFT1, TRAF3IP3, RPL26P19, QARS1, TRIM22, COMMD8, NCBP2, CTNNBL1, RPL23AP2, DEGS1, DPF2, CS, OAT, TPMT, RPL7P23, SND1, NGLY1, DDX18, RPL10A, CCT3, UBE2G2, CCT7, EPRS1, RBM5, PRCP, RTCB, SLC25A38, XRN2, PDCD6IP, IK, CD4, RPL3P7, PA2G4
PC2	AC084364.1, RPS3AP34, AC092354.1, AC008785.1, RPL19P13, FP236383.12, AC091231.1, PSME2P2, AC079448.1, GAPDHP42, AC005632.4, OR4D11, IFITM3P6, RN7SL180P, RNU4-40P, OLA1P2, AC087441.1, RPL21P126, RNU6-254P, RNA5SP78, AL359821.1, AC084036.1, MTRES1P1, FP236383.10, AC009086.1, AL109936.3, AC005631.1, SRSF3P6, SNHG22, AL133230.1, RNU1-136P, AC002073.1, GAPDHP62, AC126172.1,AL157702.1, SCARNA9, RPL21P89, AC108693.1, MIR302B, RPL22P18, SNORA59B, AC090574.1,RPS15AP5, RNU7-41P, AC013417.1, AC005046.1, PGBD4P2, RNU6-238P, PPIAP27, PSMD10P2, MIR2052, MIR518B, CR383656.3, AC022973.1, AL355309.1, AL590095.1, AC004522.1, RNU6-977P, MIR3128, NDUFS5P5, RPS15AP30,RPEP4, HSPE1P16, AC091825.1, PDCD5P1, AC068050.1, AL121890.5, LINC01578, AC055855.1, RNU6-887P, SNORA70F, RNU6-883P, SMARCE1P5, AC020910.1, AC008915.1, AC084754.1, AL136038.1, AC058791.1, AC099789.1, OOSP1P2, AC021822.1, AP004195.1, RNU6-428P, RNU6-1294P, RNU6-180P, AP003499.1, AL669983.1, RPL21P44,AL049597.1, MTND4LP26, RNU1-63P, AL161781.1, RNU6-678P, AC013267.2, LRRC37A14P, RNU2-2P, EML4, MIR524, EBLN2, NDUFB4P3
PC3	RHEBP2, RAB4B, RHEBP1, CHMP6, FKBP8, DCTN3, EGFL7, GP9, SPCS1, CCDC124, SCNM1, ANP32B, TBCB, SPINT2, PPDPF, RBCK1, LAT, ARF5, ADRM1, TMED3, C7orf50, PPP1R15A, DRAP1, CHCHD2, VAMP2, RGS3, SCN1B,CLTA, AP000547.3, TRBV28, GET3, ZNHIT1, PGAM1, SMIM10L1, MRPL28, ANAPC11, TUBA8, PSMA1, EID1, IDH2, PIN1, ATP6V1G1, PDCD6, SNAPIN, EIF1B, SWI5, ANAPC15, GPX1P1, C22orf39, WBP1, ISCU, COX4I1, SUSD3, RER1, FTH1P8, EGLN2, HMG20B, ITGA9-AS1, AP3S1, NAA10, LSM1, CFL1P4, POLR3GL, CMAS, BUD31, HMGXB4, FXYD5, TRAPPC3, H2AJ, CIRBP, NOTCH2NLC, MPST, REXO2, REX1BD, COQ4, GADD45A, VAMP8, NOSIP, TRAPPC5, ZNF655, FTH1P12, TEN1, SELENOK, TRIR, PRDX5, AC012085.1, RPL3P4, KRT18, CTSD, GTF3C6, MEA1, NDUFB10, ZMAT2, ALKBH7, AP2S1, H2AZ1, ASRGL1, HTATIP2, IGKV3D-11, SDHC
